# Mixed Compound of DCPTA and CCC Increases Maize Yield by Improving Plant Morphology and Up-Regulating Photosynthetic Capacity and Antioxidants

**DOI:** 10.1371/journal.pone.0149404

**Published:** 2016-02-12

**Authors:** Yongchao Wang, Wanrong Gu, Tenglong Xie, Lijie Li, Yang Sun, He Zhang, Jing Li, Shi Wei

**Affiliations:** 1 College of Agriculture, Northeast Agricultural University, Harbin 150030, Heilongjiang, People’s Republic of China; 2 The observation experiment station of ministry of agriculture for crop cultivation science in northeast area, Harbin 150030, Heilongjiang, People’s Republic of China; United States Department of Agriculture, UNITED STATES

## Abstract

DCPTA (2-diethylaminoethyl-3, 4-dichlorophenylether) and CCC (2-chloroethyltrimethyl- ammonium chloride) have a great effect on maize growth, but applying DCPTA individually can promote the increase of plant height, resulting in the rise of lodging percent. Plant height and lodging percent decrease in CCC-treated plants, but the accumulation of biomass reduce, resulting in yield decrease. Based on the former experiments, the performance of a mixture which contained 40 mg DCPTA and 20 mg CCC as active ingredients per liter of solution, called PCH was tested with applying 40mg/L DCPTA and 20mg/L CCC individually. Grain yield, yield components, internode characters, leaf area per plant, plant height and lodging percent as well as chlorophyll content, chlorophyll fluorescence, enzymatic antioxidants, membranous peroxide and organic osmolyte were analyzed in two years (2011 and 2012), using maize hybrid, Zhengdan 958 (ZD 958) at density of 6.75 plants m^-2^. CCC, DCPTA and PCH were sprayed on the whole plant leaves at 7 expanded leaves stage and water was used as control. Compared to control, PCH significantly increased grain yield (by 9.53% and 6.68%) from 2011 to 2012. CCC significantly decreased kernel number per ear (by 6.78% and 5.69%) and thousand kernel weight (TKW) (by 8.57% and 6.55%) from 2011 to 2012. Kernel number per ear and TKW increased in DCPTA-treated and PCH-treated plants, but showed no significant difference between them. In CCC-treated and PCH-treated plants, internode length and plant height decreased, internode diameter increased, resulting in the significant decline of lodging percent. With DCPTA application, internode diameter increased, but internode length and plant height increased at the same time, resulting in the augment of lodging percent. Bending strength and puncture strength were increased by applying different plant growth regulators (PGRs). In PCH-treated plants, bending strength and puncture strength were greater than other treatments. Compared to control, the bending strength of 3rd internode was increased by 14.47% in PCH-treated plants in 2011, increased by 18.40% in 2012, and the difference was significant. Puncture strength of 1st, 3rd and 5th internode was increased by 37.25%, 29.17% and 26.09% in 2011 and 34.04%, 25% and 23.68% in 2012, compared to control. Leaf area and dry weight per plant reduced significantly in CCC-treated plants, increased in DCPTA-treated and PCH-treated plants from 2011 to 2012. Chlorophyll content and chlorophyll fluorescence improved with CCC and DCPTA application. Due to the additive effect of DCPTA and CCC, PCH showed the significant effect on chlorophyll content and chlorophyll fluorescence. Compared to control, total enzyme activity (SOD, POD, CAT, APX and GR) and soluble protein content increased, malonaldehyde (MDA) and hydrogen peroxide (H_2_O_2_) content reduced in PCH-treated plants. The transportation of soluble sugar from leaf to kernel improved significantly at the late silking stage. The research provided the way for the further use of DCPTA and CCC into the production practice.

## Introduction

Maize (*Zea mays L*.) is one of the most important crops in the world. It can be used as food, livestock feed and bio-fuel at a global scale, and the demand for maize is growing [1, 2]. China is one of the most important maize producers in the world [3]. The total maize area was more than 36 million ha in 2013, and the total maize production was more than 218 million t [4]. Maize area and production in Heilongjiang province, Northeast China, accounted for about 22.1% and 36.3% of the total respectively. This area has many large scale national farms with mechanized crop management and high yields.

Silking stage is an important period of yield formation. At the stage, lodging, photosynthetic capacity decline and leaf senescence will restrict yield formation seriously. Lodging is a phenomenon of stems changing from upright to folding down and caused by variety of factors. It is a key factor in maize yield and some reports showed that 5–25% yield loss annually is due to lodging in America [5]. Lodging led to mutual occlusion in maize leaves, as a result, photosynthetic rate declines [6, 7]. Photosynthesis is the main way of accumulating organic matter of plant. Organic matter which is produced by photosynthesis accounts for about 95% of the total plant dry matter weight [8]. After silking, the contribution rate of photosynthesis to yield is more than 90% and it has an effect for yield formation [9]. But at the stage, leaves begin to senescence and photosynthetic ability declines, which limit the accumulation of organic matter [10]. Leaf senescence can lead to an accumulation of excess reactive oxygen species and lipid peroxides. Plants have evolved complex enzymatic and non-enzymatic antioxidant defense systems to regulate cellular oxidative damage. It is well known that superoxide dismutase (SOD), catalase (CAT), and ascorbate peroxidases (APX) are important ROS scavenging enzymes, and they have been well studied in different plants [11, 12].

Plant growth regulators (PRGs) have been introduced to control lodging recently, photosynthetic capacity decline and leaf senescence in crop [13]. Several reports had shown that jasmonates played an important role in signaling drought-induced antioxidant responses. Exogenously supplied jasmonic acid (JA) or methyl jasmonate (Me-JA) could increase the transcript levels and activities of antioxidant enzymes in plants under water stress [14]. Phosphatidic acid (PA) reduced reactive oxygen species (ROS) levels through inhibiting the production of ethylene (ETH) in leaves of spring wheat [15]. Accumulating evidence suggested a potential role of nitric oxide (NO) in regulating chlorophyll breakdown of horticultural products [16]. Nitric oxide (NO) treatment delayed the yellowing process and retards the onset of chlorophyll degradation in broccoli florets during storage at 20°C [17]. Brassinolide (BL) improved chlorophyll fluorescence (*F*_*v*_*/F*_*m*_*)*, increased chlorophyll content and seed yield under drought stress in soybean [18]. Ethephon is a plant growth regulator that inhibits stem elongation and promotes stem thickness, thereby improving plant morphological resistance to lodging [19]. Recently, compound mixture has been applied in crop production due to showing better regulatory effect and more regulatory path. The mixture of ethephon and diethyl aminoethyl hexanoate (DA-6) significantly increased maize yield, kernel number per ear and thousand-kernel weight (TKW), reduced plant height and lodging percentage, had been applied in maize production widely [19]. Several reports had shown that total grains, effective panicles, thousand kernel weight and yield increase by the application of gibberellin (GA_3_)+ 6-Benzylaminopurine (6-BA), 6-Benzylaminopurine (6-BA)+Brassinolide (BR) manifested a facilitating role of sucrose conversion and starch accumulation in rice [20].

DCPTA (2-diethylaminoethyl-3, 4-dichlorophenylether) is one of the most representative tertiary compounds, which has been used in wheat and maize, especially in horticultural crops, such as cucumber and tomato [21]. DCPTA-treated maize seedlings caused an increase of soluble sugar, which gave a good foundation for the higher dry matter accumulation and transportation [22]. DCPTA has various influences on physiological process in plants, which can regulate photosynthesis of the plants and the activity of antioxidant enzyme [23]. Some studies showed that DCPTA could speed up seedling growth, increase chlorophyll content and improve photosynthesis in different plants, which indicated that DCPTA participated in regulation of photosynthetic reaction [24].

Chlorocholine chloride (2-chloro-N, N, N-trimethylethanaminium chloride; CCC; CAS No. 50-29-3) is an antigibberellin growth retardant, with its mechanism based on the restraint of gibberellins biosynthesis in plant tissues [25]. It was well known that CCC could reduce the growth of stems, leaf area index and crop growth rate [26], decrease lodging but also slightly reduce yield [27]. CCC decreased the growth of stems, leaves, and stolons but promoted tuberization in potato [28]. In addition, methanolic extracts from CCC-treated calli and leaves showed significant increment in antioxidant activity as determined by standard DPPH (1,1-diphenyl-2-picryl-hydrazyl), ABTS (2,2'-Azinobis-(3-ethylbenzthiazoline-6-sul p-honate)), and hydroxyl radical scavenging assays.

DCPTA can increase yield but it results in taller stems, increases the risk of lodging in maize. CCC can make stems shorter and stronger but also slightly decreasing yield. PCH has been used in maize production in northeast of China. Limited information is yet available on the effectiveness of PCH. The aim of this study was to investigate whether combining CCC with DCPTA could offset the yield disadvantages of CCC and thus achieve higher yield in combination with lower risk of lodging, higher photosynthetic capacity and antioxidant enzyme activity in maize.

## Materials and Methods

### Experimental site and cultivar

Field trials were conducted from 2011 to 2012 at the Experimental Station of Northeast Agricultural University, Harbin (126°73’E, 45°73’N), Heilongjiang province, China. The soil was a typical black soil (typical hapludoll in USDA soil taxonomy), characterized by a deep, high organic matter content. The soil fertility level was determined before sown (Table 1). The climate was a temperate continental monsoon in the region. The rainfall was variable with greater distribution in July and August. Maize was planted at mid-April and harvested at early October. Weather data in 2011 and 2012 are given in Table 2.

**Table 1:** Background data of productivity and soil fertility of the experimental fields studied from 2011 to 2012.

Year	pH	Water content(%)	Organic matter (g/kg)	Total N(mg/kg)	Available P(mg/kg)	Available K(mg/kg)
**2011**	6.50	20.32	15.64	1.57	53.26	112.48
**2012**	6.76	18.94	14.83	1.62	51.82	110.34

Data were collected from 0 to 20 cm soil samples in early spring before irrigation each year.

**Table 2:** Daily mean values of weather variables at experimental site during each of six months of the maize growing season from 2011 to 2012.

Month	Average temperature (°C)		Precipitation (mm)		Sunshine hours (h)		Maximum instantaneous wind speed (ms^-1^)	
	2011	2012	2011	2012	2011	2012	2011	2012
**April**	7.6	7.8	26.9	38.1	235.9	183.0	10.97	10.55
**May**	14.8	16.4	79.4	28.8	196.4	230.5	9.62	8.84
**June**	21.6	21.3	41.3	154.7	231.3	167.5	8.21	8.11
**July**	24.5	23.9	143.3	129.9	196.9	135.7	7.42	7.16
**August**	22.7	21.8	80.9	214.7	238.9	203.8	6.89	7.22
**September**	15.0	16.4	29.3	81.9	249.0	155.8	8.11	6.94
Total*	17.7	17.9	401.1	648.1	1348.4	1076.3	8.54	8.13

*Precipitation and sunshine are monthly sums, and temperature is a monthly mean of daily means, and maximum instantaneous wind speed is a monthly mean of the daily maximum values. The date is from China meteorological data network (http://data.cma.gov.cn/).

A high-yielding commercial cultivar, Zhengdan 958 (ZD 958) was used for two years experiments, provided by Beijing Doneed Co.,Ltd. The seeds (percentage germination≥85%) were treated with Tebuconazole (triazole fungicide) by the Rainsun Agrochemical Company Ltd., Qingdao, Shandong. In the study area, the growth periods and active accumulated temperature of ZD958 were about 128 days and above 2850°C respectively.

### Experiment design

Maize was hand-sown at 7cm depth and 70cm row distance on April 28 in 2011and on April 29 in 2012. Seeds were coated to prevent pests and fungi. In one pilot area, four treatments were set, application with water, CCC, DCPTA and PCH under the plant density of 6.75 plants m^-2^. The area was 56m^2^ for each treatment, set up 8 ridges, the length and weight of each ridge was 10m and 0.7m respectively. A randomized block design with three replications was used for the study.

The applied concentrations of DCPTA and CCC were 40 mg/L and 20 mg/L respectively. PCH was made up DCPTA (Zhengzhou Zheng Shi Chemical Co., Ltd. China), CCC (Sigma-Aldrich Co. LLC, St. Louis, MO, USA) and distilled water, containing 40 mg DCPTA and 20 mg CCC as active ingredients per liter of PCH solution. PCH, CCC and DCPTA were manually applied on both sides of leaves by a back-pack sprayer at a rate of 680 L ha^-1^ (about 10 ml per plant) at the stage of 7 expanded leaves stage (V7) on June 17 in 2011 and June 19 in 2012 of the afternoon (16:00–19:00), control plants were treated with water. During processing and after, growing environment was good and no have inclement weather. The precipitation was all 0 mm, the average temperature was 24°C and 22°C, the average wind speed was 1.8 ms^-1^ and 1.3 ms^-1^, the sunshine hours was 11.8 h and 11.4 h on June 17 of 2011 and June 19 of 2012 respectively. The type of back-pack sprayer is ‘gongnong-16’, and the main structures includes switch, spray lance, nozzle, spray tank, piston rod, pump cylinder, air plenum, outlet valve, inlet valve and hand shank. The carrier volume of spray tank is 50 L; chamber pressure is about 3.92×10^−3^ Pa after pressurizing. The aperture of nozzle plate of 1.5 mm can produce droplets having a diameter of 350μm, accompanying flowing speed of 15.2 ml s^-1^.

### Field management

Fertilizer application followed high yield practice with a base fertilizer gift of 75 kg N ha^-1^, 75 kg P_2_O_5_ ha^-1^, 90 kg K_2_O ha^-1^, and a top dressing with 150 kg ha^-1^ of urea at 7 expanded leaves stage and 75 kg ha^-1^(46% N) at tassel stage. With manual thinning out the maize plant, one plant was remained per sowing point at the two-leaf stage. Other management practices, including insect and weed control were conducted according to local agronomic practices unless otherwise indicated.

### Measurements

Maize ear leaves were measured and sampled at 10, 20, 30, 40, 50 days after silking. In one pilot area, five plants were randomly selected and listed for measuring chlorophyll fluorescence parameters in each treatment. Five ear leaves were cut, treated with liquid nitrogen and stored in -80°C for determination of SOD, POD, CAT, APX and GR activity, MDA, H_2_O_2_, soluble protein and soluble sugar content in each treatment. Chlorophyll content was measured with fresh leaves. First sampling was 20th July and 15th July, 2011 and 2012. The date (S1 File) was used for analysis of variance.

### Yield and agronomic characters

To determine yield, maize ears (28 square meters) of each treatment were hand harvested from each plot at crop maturity on 25th and 27th September, 2011 and 2012. All harvested areas were surrounded by 5 guard rows. Grain yield and thousand kernel weight (TKW) were converted to yield using a fixed grain water content of 14%. Ear size (ear length and ear diameter), kernel number per ear and TKW were measured on 10 randomly selected ears in each treatment. The date (S2 File) was used for analysis of variance.

To determine agronomic characters (plant height, internode length, internode diameter, stem strength, leaf area per plant, leaf dry weight per plant), 5 plants of each treatment were selected randomly at 29th and 24th August which is 50 days after silking, 2011 and 2012(Milk stage). Plant height, and internode length was measured with meter stick that minimum scale is 0.1cm. Internode diameter was measured with calipers. Stem strength was measured with stem strength tester (AWOS-SL04). Due to bending strength of 1st internode exceed the measuring range of stem strength tester, 3rd and 5th internode were used for data analysis. Leaf area was (leaf length ×leaf width) ×0.75. Leaf was dried to constant weight and measured the dry weight.

All plants of each treatment in 28 m^2^ in each plot were evaluated at milk stage (R3) (29th August) and subsequently at dough (R4) stage (25th September) in both years to determine the percentage of lodged plants. If the plant stem angle with the vertical at the basis was greater than 30°, we classified a plant as lodged. Lodging at milk stage was minimal (<3%) in both years and mainly caused by field management. In 2012, all plants had lodged in all treatment plots due to a typhoon (maximum wind speeds of 18 m s^-1^) on 29th August, and they did not recover. Experimental Station of Northeast Agricultural University, lodging was uniformly 100% at dough stage in 2012. In the analysis, we focus on lodging effect at dough stage in 2011. The date (S3 File) was used for analysis of variance.

### Photosynthetic characters

Ear leaf that had been removed the veins was used to determine the chlorophyll content. The leaves were cut, mixed and weighed 0.2g, soaked for 72 hours at 4°C by 10ml 80% acetone in dark. The absorbance values of extracting solution were determined with UV-1601 UV-spectrophotometer by colorimetric method at wavelength of 649nm and 665nm [29].

Chlorophyll fluorescence parameters of the middle part of ear leaves were determined with PAM-2500 chlorophyll fluorescence analyzer (WALZ, Germany) between 9:00 and 12:00 in sunny. After a 20 min dark adaptation period, the initial (*F*_*0*_) and maximum fluorescence (*F*_*m*_) were determined. Light intensity was set 600 μmolm^-2^s^-1^ for determining maximum fluorescence (*F*_*m*_*'*), initial fluorescence (*F*_*0*_*'*), steady-state fluorescence (*F*_*s*_). Maximal photochemical efficiency of *PSII F*_*v*_*/F*_*m*_ = (*F*_*m*_*-F*_*0*_)/*F*_*m*_, potential photochemical efficiency *F*_*v*_*/F*_*0*_ = (*F*_*v*_*/F*_*m*_)/ (1-*F*_*v*_*/F*_*m*_), actual photochemical efficiency of PSII in the light Y (II) = (*F*_*m*_*'*-*F*_*s*_)/*F*_*m*_*'* [30].

### Antioxidant enzyme activity

SOD activity was determined according to Giannopolitis [31]. 20 μL enzyme solution was drawn and mixed with 3 mL SOD reaction solution (pH 7.8 phosphate buffer 1.5 mL, 750 mol L^-1^ NBT 0.3 mL, 130 mmol L^-1^ Met 0.3 mL, 20 mol L^-1^ FD 0.3 mL, 100 mol L^-1^ EDTA-Na_2_ 0.3 mL, distilled water 0.3 mL). Control and enzyme solution were placed for 30 min in 4000 lux light. The blank was placed in dark for zero, compared in 560nm.

POD activity was determined according to Hernandez [32]. 20 μL enzyme solution was drawn and mixed with 3mL POD reaction solution (1.4 μL guaiacol, 0.85 μL 30% H_2_O_2_ and 0.1mol L^-1^ pH 6.0 phosphate buffer).The absorbance values were recorded once every 30 s in 470 nm.

CAT activity was assayed as a decrease in absorbance at 240 nm for 1 min following the decomposition of H_2_O_2_ according to Change and Maehly [33]. The reaction mixture contained 50 mM phosphate buffer (pH 7.0) and 15 mM H_2_O_2_.

APX activity was determined according to Nakano and Asada [34]. The assay mixture consisted of 0.5 mM ASA, 0.1 mM H_2_O_2_, 0.1 mM EDTA, 50 mM sodium phosphate buffer (pH 7.0), and 0.15 ml enzyme extract.

GR activity was assayed as described by Foyer and Halliwell [35]. The oxidized GSH (GSSG)-dependent oxidation of NADPH was followed at 340 nm in a 1mL reaction mixture containing 100 mM sodium phosphate buffer (pH 7.8), 0.5 mM GSSG, 50 μL extract, and 0.1 mM NADPH.

MDA content was measured as 2 mL enzyme solution was drawn and mixed with 0.67% TBA 2 mL, than water-bath heating for 30 min in 100°C, centrifuged after cooling down. The supernatant was determined respectively in 450 nm, 532 nm and 600 nm [36]. The H_2_O_2_ content was measured using the method of Xie and others [37].

Soluble protein concentration was measured with coomassie brilliant blue G-250 staining [38]. Soluble sugars were extracted and analyzed according to Ci and others [39].

### Statistical analysis

Experimental data were expressed as mean with standard deviation. Statistical analysis was performed using SPSS 15.0 and Excel 2007 and all means were carried out using the LSD Fischer test at a significance level of p<0.05.

## Results

### Ear size

Ear size is associated with grain full extent. Ear size was significantly affected by PGRs but not by year. The interaction between year and PGRs was not significant (Table 3). Compared to control, the ear length was decreased by 7.94% and 9.28% from 2011 to 2012 with application CCC. But, it was increased by 2.47%, 0.5% and 1.02%, 0.56% from 2011 to 2012 with DCPTA and PCH spraying (Table 4).

**Table 3:** Results of ANOVA on the effects of year (Y), plant growth regulators (PGRs) on maize grain yield, yield components and ear shape.

Effect	df^d^	Yield	Thousand kernels weight	Kernel numbers per ear	Ear length	Ear diameter
**Year**	1	19.729^a^	60.214^b^	72.698^b^	0.078^c^	1.931^c^
**PGRs**	2	12.632^a^	34.648^b^	16.227^b^	10.084^a^	19.323^b^
**Year×PGRs**	2	0.816^c^	0.669^c^	0.46^c^	0.079^c^	0.127^c^
**Error**	32					
**Total variation**	39					

^a^ *F* values and significance levels at P < 0.05.

^b^ *F* values and significance levels at P < 0.01.

^c^ *F* values and significance levels at P≥0.05.

^d^ The df are given for hypothesis (error). Grain yield was calculated with 14% water content. The applied concentrations of DCPTA and CCC were 40 mg/L and 20 mg/L respectively. PCH was made up DCPTA, CCC and distilled water, containing 40 mg DCPTA and 20 mg CCC as active ingredients per liter of PCH solution, which were applied at a rate of 675 L ha^-1^ at the stage of 7 expanded leaves.

**Table 4:** Effects of different plant growth regulators on maize grain yield (t ha^-1^) and yield components from 2011 to 2012.

Year	Treatment	Ear length(cm)	Ear diameter(cm)	Kernel number per ear	Thousand kernel weight(g)	Yield(t ha^-1^)
**2011**	**Control**	19.40±0.99ab	5.24±0.09b	590±20.00a	315±10.00bc	12.59±0.58b
	**CCC**	17.86±0.79b	5.16±0.16b	550±15.00b	300±14.00c	11.55±0.92b
	**DCPTA**	19.88±0.85a	5.62±0.21a	622±22.00a	321±3.50ab	13.67±0.78a
	**PCH**	19.50±1.29ab	5.40±0.09ab	600±25.00a	337±7.00a	13.79±0.78a
**2012**	**Control**	19.62±0.92a	5.32±0.19b	650±9.00a	290±9.00b	11.83±0.83b
	**CCC**	17.80±0.76b	5.18±0.10b	613±22.00b	271±7.00c	10.75±0.74b
	**DCPTA**	19.82±1.07a	5.72±0.21a	665±19.00a	301±10.00b	11.92±1.01ab
	**PCH**	19.73±0.43a	5.48±0.17ab	658±27.00a	319±8.50a	12.62±0.61a

Values are mean ± SD. Values with the same letters in a column are not significantly different at P< 0.05 (LSD test) in same year

Compared to control, the ear diameter was decreased by 1.34% and 2.44% from 2011 to 2012, respectively, with application CCC. It showed promotion of 7.27% in 2011 and 7.52% in 2012 with application DCPTA. Similarly, ear diameter was increased by 3.44% and 3.20% from 2011 to 2012, respectively, in PCH-treated plants. The increase of ear length and ear diameter were inhibited in CCC-treated plants, but showed the opposite effect in DCPTA-treated plants. With application PCH, ear size showed a promoting effect due to the inhibition of CCC was weakened by DCPTA in PCH (Table 4).

### Grain yield and yield components

Grain yield is mainly composed of thousand kernel weight (TKW) and kernel number per ear. Kernel number per ear was significantly affected by year and PGRs, but the interaction between year and PGRs was not significant (Table 3). With DCPTA application, kernel number per ear was increased by 5.43% and 2.31% from 2011 to 2012, respectively, compared to control. PCH-treated plants showed the same effect in kernel number per ear, increased by 1.69% and 1.23% from 2011 to 2012, but it decreased in CCC-treated plants (Table 4).

TKW was significantly affected by year and PGRs, but the interaction between year and PGRs was not significant (Table 3). TKW was higher than control in PCH and DCPTA-treated plants. It was lower than control in CCC-treated plants from 2011 to 2012. Compared to CCC and DCPTA, TKW was increased by 12.33%, 4.98% and 17.71%, 5.98% from 2011 to 2012, respectively, with application PCH, the difference was significant (Table 4).

Grain yield was significantly affected by year and PGRs, but the interaction between year and PGRs was not significant (Table 3). Grain yield of control, CCC, DCPTA and PCH were 12.59, 11.55, 13.67, 13.79 t ha^-1^ in 2011 and 11.83, 10.75, 11.92, 12.62 t ha^-1^ in 2012. Compared to control, grain yield was increased by 9.53% and 6.68% from 2011 to 2012, respectively, with PCH application. Over the two years, grain yield of PCH was higher than another due to higher TKW, even though PCH-treated plants were not the most ear size and kernel number per ear. Grain yield and TKW in 2012 were lower than in 2011 due to lodging after a typhoon at dough stage (Table 4).

### Internode length and internode diameter

Stem trait is an important factor of affecting the lodging resistance. With different PGRs application, 1st, 3th and 5th internode length had a change in maize. CCC-treated plants showed the shortest internode length in 1st, 3th and 5th internode which were decreased by 12.58%, 10.68%, and 7.98% in 2011, 9.19%, 14.49% and 9.94% in 2012, respectively, compared to control. DCPTA-treated plants had the longest internode length in 1st, 3th and 5th internode which were increased by 4.95%, 4.01% and 8.54% in 2011, 6.79%, 11.04% and 16.91% in 2012, respectively, compared to control. PCH had the same effect with CCC on internode length, made 1st, 3th and 5th internode length show a 9.50%, 7.31% and 3.52% reduction in 2011, 2.66%, 15.09% and 9.18% reduction in 2012, respectively. The effect of PCH shortened internode length was inferior to CCC due to DCPTA had the effect of promoting internode growth (Table 5).

**Table 5:** Effects of different plant growth regulator on internode length (cm) and internode diameter (mm) from 2011 to 2012.

Year	Treatment	Internode length (cm)			Internode diameter (mm)		
		1	3	5	1	3	5
**2011**	**Control**	7.47±0.39ab	13.95±0.88ab	18.16±1.00ab	28.02±0.67c	25.66±1.84c	22.68±2.03b
	**CCC**	6.53±0.50b	12.46±0.40c	16.71±0.71b	32.72±2.23ab	30.94±2.96ab	26.84±1.52a
	**DCPTA**	7.84±0.79a	14.51±0.50a	19.71±0.74a	29.64±1.02bc	27.82±1.18bc	25.02±1.26ab
	**PCH**	6.76±0.71b	12.93±0.90bc	17.52±0.88b	34.98±2.55a	31.96±2.30a	27.22±0.55a
**2012**	**Control**	7.51±0.5ab	13.32±0.57b	18.51±0.50b	28.80±1.49c	26.96±1.63b	24.74±1.85b
	**CCC**	6.82±0.79b	11.39±0.29c	16.77±0.85c	33.02±0.92ab	29.96±1.80ab	27.34±1.35ab
	**DCPTA**	8.02±0.61a	14.79±0.71a	21.64±0.97a	30.10±2.79bc	27.22±1.99b	25.90±2.27ab
	**PCH**	7.31±0.29ab	11.31±0.66c	16.81±0.80c	35.42±2.15a	31.38±2.39a	28.26±1.06a

Values are mean ± SD. Values with the same letters in a column are not significantly different at P < 0.05 (LSD test) in same year.

Compared to control, internode diameter was increased in varying degrees in three PGRs-treated plants. PCH-treated plants had the most significant effect on 1st, 3th and 5th internode diameter and they were increased by 24.83%, 24.55% and 20.02% in 2011, 22.94%, 16.36% and 14.27% in 2012, respectively. Over the two years, the promoting effect of PCH on internode diameter was weakened gradually from 1st to 5th internode. PCH-treated plants had the most thick internode diameter than the other plants due to the additive effect of CCC and DCPTA (Table 5).

### Internode strength

Bending strength and puncture strength are important traits of lodging resistance in stem and their strength can be increased by using different PGRs. Compared to control, the bending strength of 3rd internode was increased by 14.47% in PCH-treated plants in 2011, increased by 18.40% in 2012, the difference was significant. The bending strength of 3rd internode was increased in CCC and DCPTA-treated plants, compared to control, and the difference was not significant in 2011. The bending strength of 5th internode was increased by using PGRs, the best effect appeared in PCH-treated plants and the difference was significant, compared to control from 2011 to 2012 (Table 6).

**Table 6:** Effects of different plant growth regulator on stem strength (N) from 2011 to 2012.

Year	Treatment	Bending strength (N)		Puncture strength (N)		
		3	5	1	3	5
**2011**	**Control**	228±9.9b	143±11.9c	51±4.0c	48±2.9b	46±2.9c
	**CCC**	243±13.0ab	168±6.1ab	62±2.1b	56±6.1b	53±4.0b
	**DCPTA**	236±6.1b	160±9.0bc	54±4.0c	51±5.0bc	48±2.1bc
	**PCH**	261±11.0a	177±10.0a	70±6.1a	62±5.0a	58±2.9a
**2012**	**Control**	212±10.0c	139±9.0b	47±4.0c	40±5.0c	38±2.9b
	**CCC**	241±10.0ab	151±6.1b	57±5.0ab	49±5.0ab	43±2.1ab
	**DCPTA**	229±9.0bc	144±4.0b	53±2.9bc	44±3.1bc	40±4.0b
	**PCH**	251±8.5a	169±7.1a	63±4.0a	50±3.3a	47±4.2a

Values are mean ± SD. Values with the same letters in a column are not significantly different at P<0.05 (LSD test) in same year.

In PCH-treated plants, puncture strength of 1st, 3rd and 5th internode was increased by 37.25%, 29.17% and 26.09% in 2011 and 34.04%, 25% and 23.68% in 2012, compared to control, the difference was significant. In CCC-treated plants, puncture strength of 1st, 3rd and 5th internode was increased by 21.57%, 16.67% and 15.21% in 2011 and 21.28%, 20% and 13.16% in 2012, compared to control. In DCPTA-treated plants, puncture strength of 1st, 3rd and 5th internode was increased by 9.80%, 6.25% and 4.35% in 2011 and 12.77%, 10% and 5.26% in 2012, compared to control, the difference was not significant. The data showed the effect of different PGRs on puncture strength was weakened sequentially from 1st internode to 5th internode, PCH showed the best effect on puncture strength (Table 6).

### Leaf area and leaf dry weight per plant

With different PGRs application, leaf area per plant and leaf dry weight per plant showed significantly different. Compared to control, leaf area per plant was reduced to 12.49% and 14.18% from 2011 to 2012, respectively, in CCC-treated plants. DCPTA and PCH-treated plants showed 9.81%, 0.1% and 11.51%, 1.11% increase from 2011 to 2012, respectively. Compared to control, DCPTA-treated plants showed a significant difference (Table 7).

**Table 7:** Effects of different plant growth regulator on leaf area per plant (cm^2^) and leaf dry weight per plant (g) from 2011 to 2012.

Year	Treatment	Leaf area per plant(cm^2^)	Leaf dry weight per plant(g)
**2011**	**Control**	6974±246b	50.25±3.01b
	**CCC**	6103±159c	43.2±1.44c
	**DCPTA**	7658±111a	57.45±2.17a
	**PCH**	6983±163b	52.13±1.68b
**2012**	**Control**	8251±118b	60.72±3.41bc
	**CCC**	7081±166c	54.88±3.32c
	**DCPTA**	9201±102a	69.21±2.75a
	**PCH**	8343±69b	63.99±3.01ab

Values are mean ± SD. Values with the same letters in a column are not significantly different at P<0.05 (LSD test) in same year.

Compared to control, the accumulation of dry matter was inhibited in leaf in CCC-treated plants, the difference was significant in 2011, but was not in 2012. The accumulation of dry matter was promoted in leaf in DCPTA-treated plants and the difference was significant in two years. Due to DCPTA weakened inhibitory effect of CCC, leaf dry weight per plant in PCH-treated plants was higher than control and showed a 3.74% and 5.39% increase from 2011 to 2012, but the different was not significant (Table 7).

### Plant height and lodging

**Fig 1:**
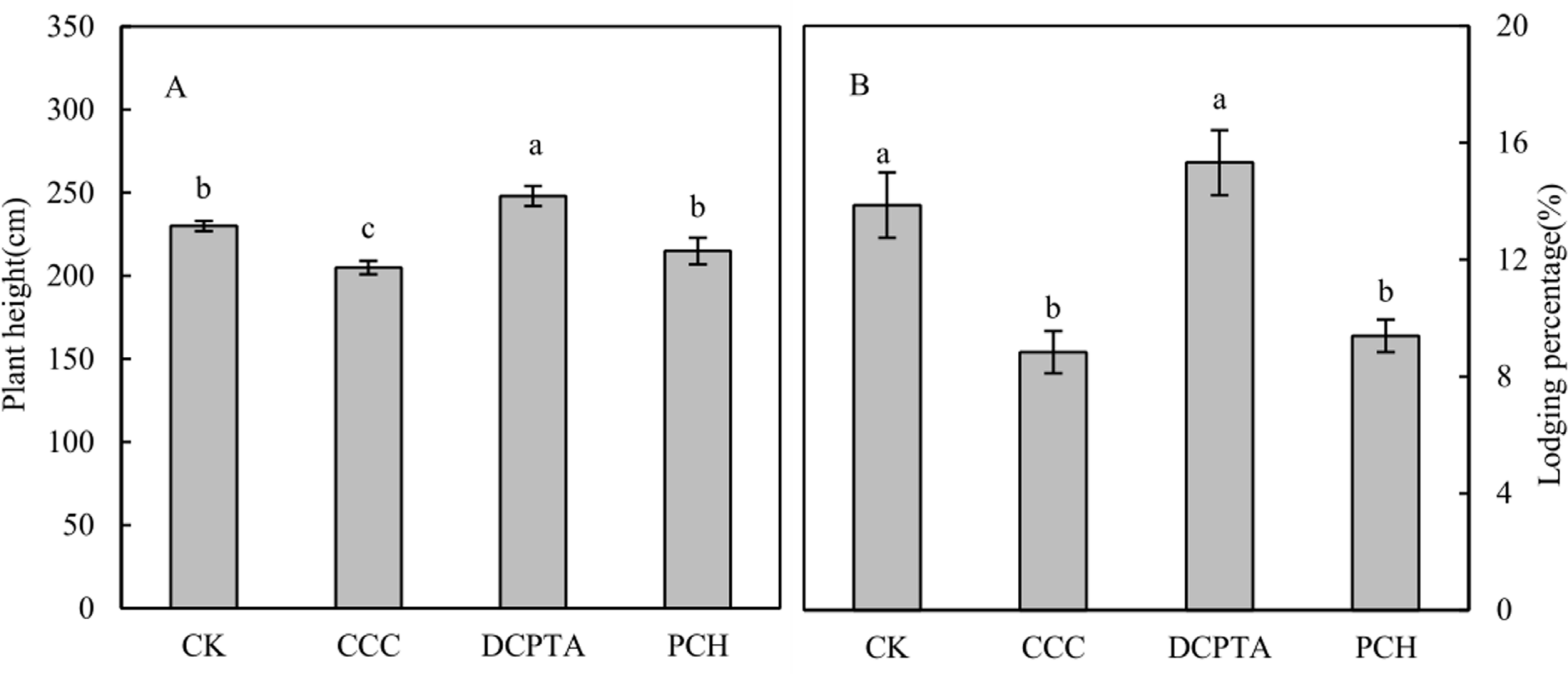
Effect of different plant growth regulators on plant heigth and lodging percengage in 2011. (A) Plant height. (B) Lodging percentage.Values are mean±SD. Same small letter on the bar indicates no significant difference at P<0.05 (LSD test).

Plant height was decreased 1.12 and 1.07-fold in CCC and PCH-treated plants, increased 1.08-fold in DCPTA-treated plants, compared to control. The difference was not significant between control and PCH due to CCC inhibited growth-promoting effect of DCPTA (Fig 1A). Lodging percentage was decreased 1.57 and 1.47-fold in CCC and PCH-treated plants, increased 1.1-fold in DCPTA-treated plants, compared to control. The difference was not significant between CCC and PCH, and was significant between PCH and control (Fig 1B).

### Chlorophyll content

**Fig 2:**
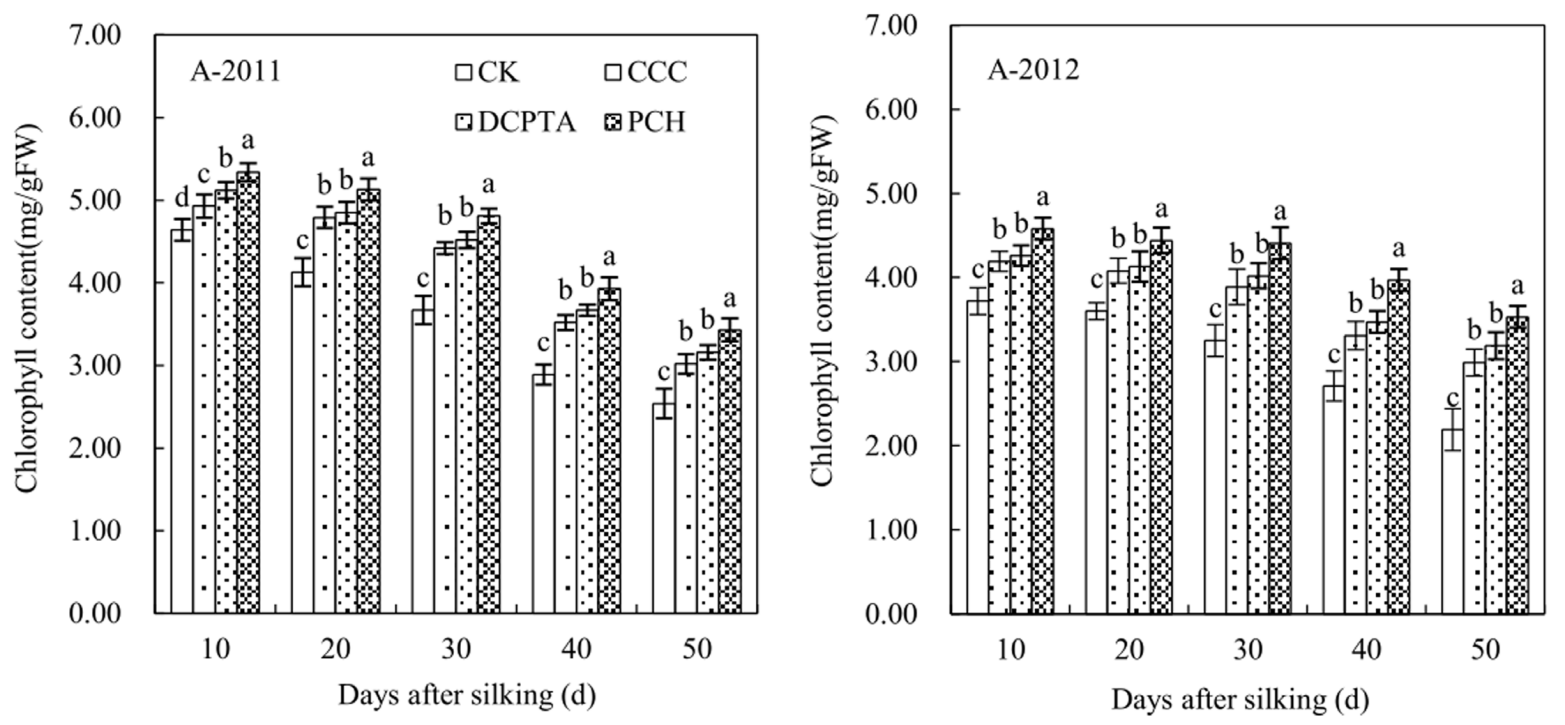
Effect of different plant growth regulators on chlorophyll content from 2011 to 2012. Values are mean±SD. Same small letter on the bar indicates no significant difference at P<0.05 (LSD test).

Chlorophyll is the main pigment of photosynthesis, and its content represents the photosynthetic capacity. Chlorophyll content was significantly affected by year, PGRs and sampling time. The interaction between year and sampling time was significant (Table 8). With the increase in days, chlorophyll content was gradually reduction. PGRs-treated plants were no significantly change from 10 to 30 days after silking, showed a significantly change after 30 days from 2011 to 2012. Compared to control, chlorophyll content increased significantly with three PGRs application, PCH-treated plants showed the best effect. Chlorophyll content of 50 days after silking increased 1.35 and 1.61-fold from 2011 to 2012, respectively, compared to control (Fig 2).

**Table 8:** Results of ANOVA on the effects of year (Y), plant growth regulator (PGRs) and sampling time (T) on chlorophyll content, chlorophyll fluorescence, enzymatic activity and lipid peroxidation.

Effect	df	Chlorophyll content	*F*_*0*_	*F*_*v*_*/F*_*m*_	*Y (II)*	*qP*	*qN*	SOD	POD	CAT	APX	GR	MDA content	H_2_O_2_ content
Y	1	423.41^b^	73.50^b^	18.41^b^	1.29^c^	8.18^b^	28.01^b^	999.23^b^	3.64^c^	64.71^b^	1233^b^	4.54^a^	1162^b^	2156^b^
PGRs	3	421.68^b^	245.53^b^	131.44^b^	179.39^b^	66.99^b^	91.88^b^	138.68^b^	76.58^b^	1.31^c^	212.72^b^	215.72^b^	117.01^b^	298.27^b^
T	4	830.08^b^	841.71^b^	232.59^b^	309.12^b^	216.81^b^	255.65^b^	511.73^b^	298.73^b^	104.55^b^	490.64^b^	763.16^b^	1831^b^	1268^b^
Y×PGRs	3	2.30^c^	0.42^c^	2.01^c^	12.5^b^	2.22^c^	1.68^c^	12.42^b^	7.50^b^	1.59^c^	18.33^b^	0.83^c^	0.42^c^	46.89^b^
Y×T	4	49.28^b^	1.61^c^	2.45^c^	7.61^b^	1.89^c^	2.56^c^	5.95^b^	32.37^b^	2.97^a^	9.25^b^	1.29^c^	15.99^b^	28.99^b^
PGRs×T	12	1.712^c^	1.76^c^	0.66^c^	1.83^a^	1.64^c^	2.24^c^	4.30^b^	5.15^b^	1.68^c^	3.44^a^	1.16^c^	16.69^b^	0.37^c^
Y×PGRs×T	12	1.541^c^	1.01^c^	0.37^c^	1.56^c^	2.72^c^	1.65^c^	2.16^c^	2.41^b^	1.73^c^	1.52^c^	0.75^c^	1.61^c^	6.95^b^
Error	160													
Total variation	199													

^a^*F* values and significance levels at P < 0.05.

^b^ *F* values and significance levels at P < 0.01.

^c^ *F* values and significance levels at P≥0.05.

### Chlorophyll fluorescence

**Fig 3:**
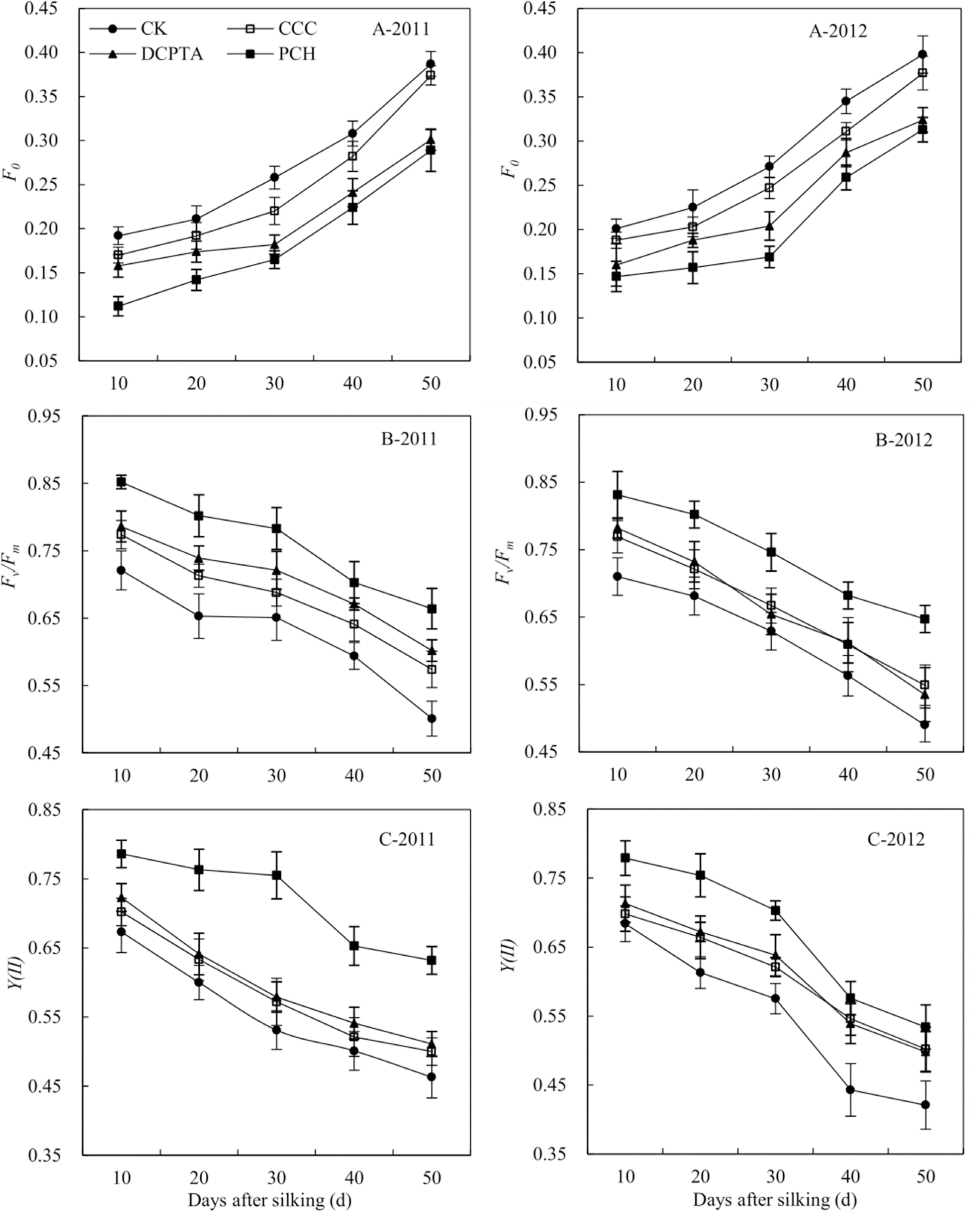
Effect of different plant growth regulators on chlorophyll fluorescence from 2011 to 2012. (A) Primary fluorescence, *F*_*o*_. (B) Maximum quantum efficiency of *PSII* photochemistry, *F*_*v*_*/F*_*m*_. (C) Actual quantum yield of *PSII* in light, *Y(II)*. Values are mean±SD.

Primary fluorescence (*F*_*o*_) was significantly affected by year, PGRs and sampling time. The interaction of various factors was not significant (Table 8). With the increased in days, *F*_*o*_ vaule was rose. Compared to control, *F*_*o*_ vaule was reduced in PGRs-treated plants and PCH-treated plants had the most significant effect, reduced 27.65% and 19.54% at 50 days after silking from 2011 to 2012, respectively (Fig 3A).

Maximum quantum efficiency (*F*_*v*_*/F*_*m*_) vaule was significantly affected by year, PGRs and sampling time. The interaction of various factors was not significant (Table 8). With the increased in days, *F*_*v*_*/F*_*m*_ vaule were decreased gradually. Compared to control, *F*_*v*_*/F*_*m*_ vaule was increased in different PGRs-treated plants and there was no significant difference between CCC-treated plants and DCPTA-treated plants from 2011 to 2012. PCH showed the best effect on enhancing *F*_*v*_*/F*_*m*_ vaule and slowing the rate of decline. *F*_*v*_*/F*_*m*_ vaule of PCH was 1.33 times and 1.32 times greater than control at 50 days after silking from 2011 to 2012, respectively, the difference was significant (Fig 3B).

*Y(II)* was significantly affected by PGRs and sampling time. The interaction of various factors was significant except between three factors (Table 8). With the increased in days, *Y(II)* were decreased gradually. With different PGRs application, *Y(II)* was increased, compared to control. PCH showed the best effect on slowing the rate of decline. Compare to control, CCC and DCPTA, *Y(II)* was increased to 37%, 26% and 24% at 50 days after silking in 2011, increased to 27%, 6% and 7% at 50 days after silking in 2012 in PCH-treated plants (Fig 3C).

**Fig 4:**
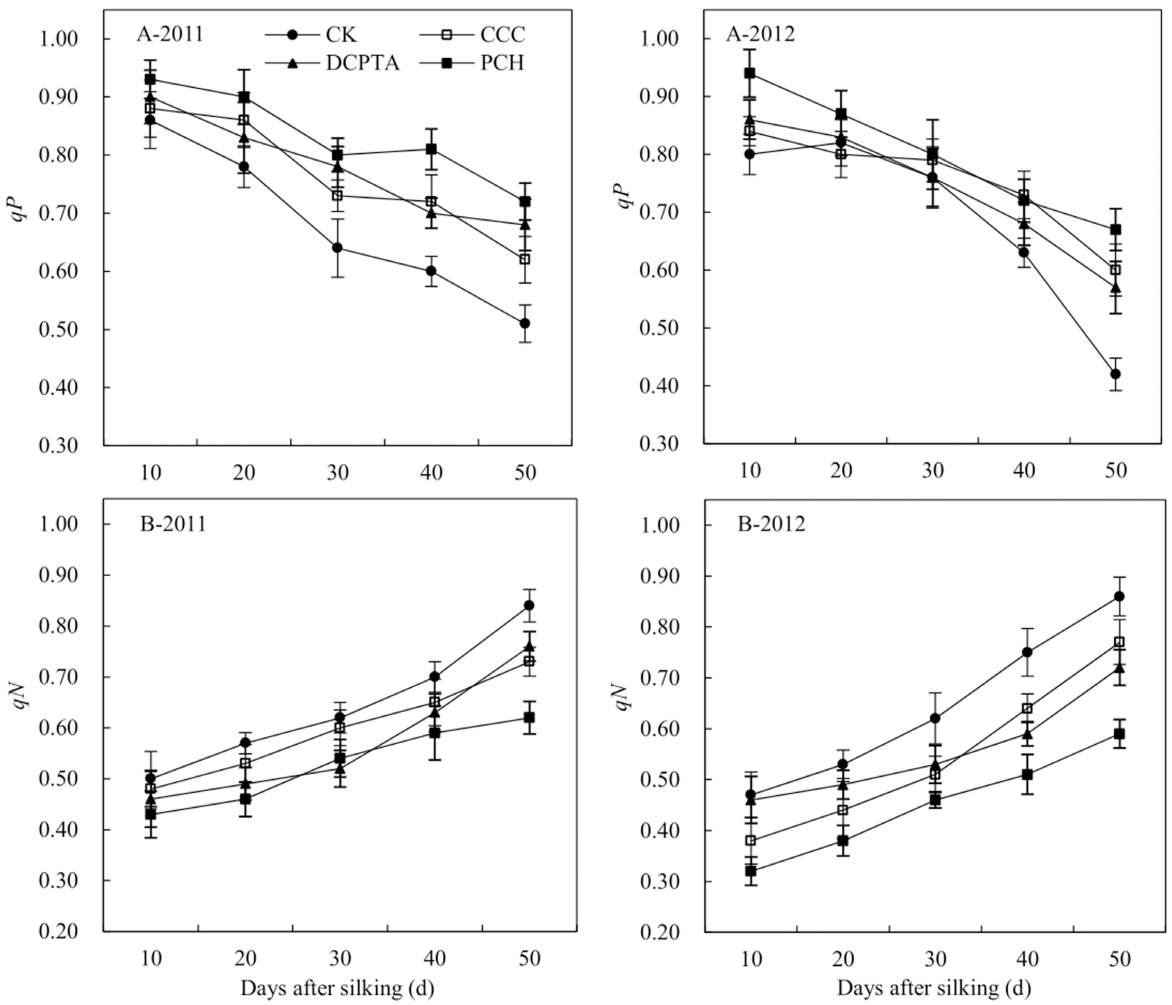
Effect of different plant growth regulators on chlorophyll fluorescence from 2011 to 2012. (A) Photochemical quenching coefficient, *qP*. (B) Non-photochemical quenching coefficient, *qN*. Values are mean±SD.

Photochemical quenching (*qP*) and non-photochemical quenching (*qN*) were significantly affected by year, PGRs and sampling time. The interaction of various factors was not significant (Table 8). From 10 to 50 days after silking, *qP* showed a downward trend and *qN* showed an upward trend from 2011 to 2012 (Fig 4). With different PGRs application, the *qP* was increased from 2011 to 2012, compared to control. PCH-treated plants showed the highest *qP* and it was higher 5.88%, 16.13% and 41.18% than DCPTA, CCC and control at 50 days after silking in 2011(Fig 4A-2011). The *qN* was decreased in PGRs-treated plants from 2011 to 2012, compare to control (Fig 4B). The *qN* was lowest in PCH-treated plants at 50 days after silking in 2012, showed a 31.40%, 23.38% and 18.06% reduction, compared to control, CCC and DCPTA (Fig 4B-2012).

### Enzymatic antioxidants

**Fig 5:**
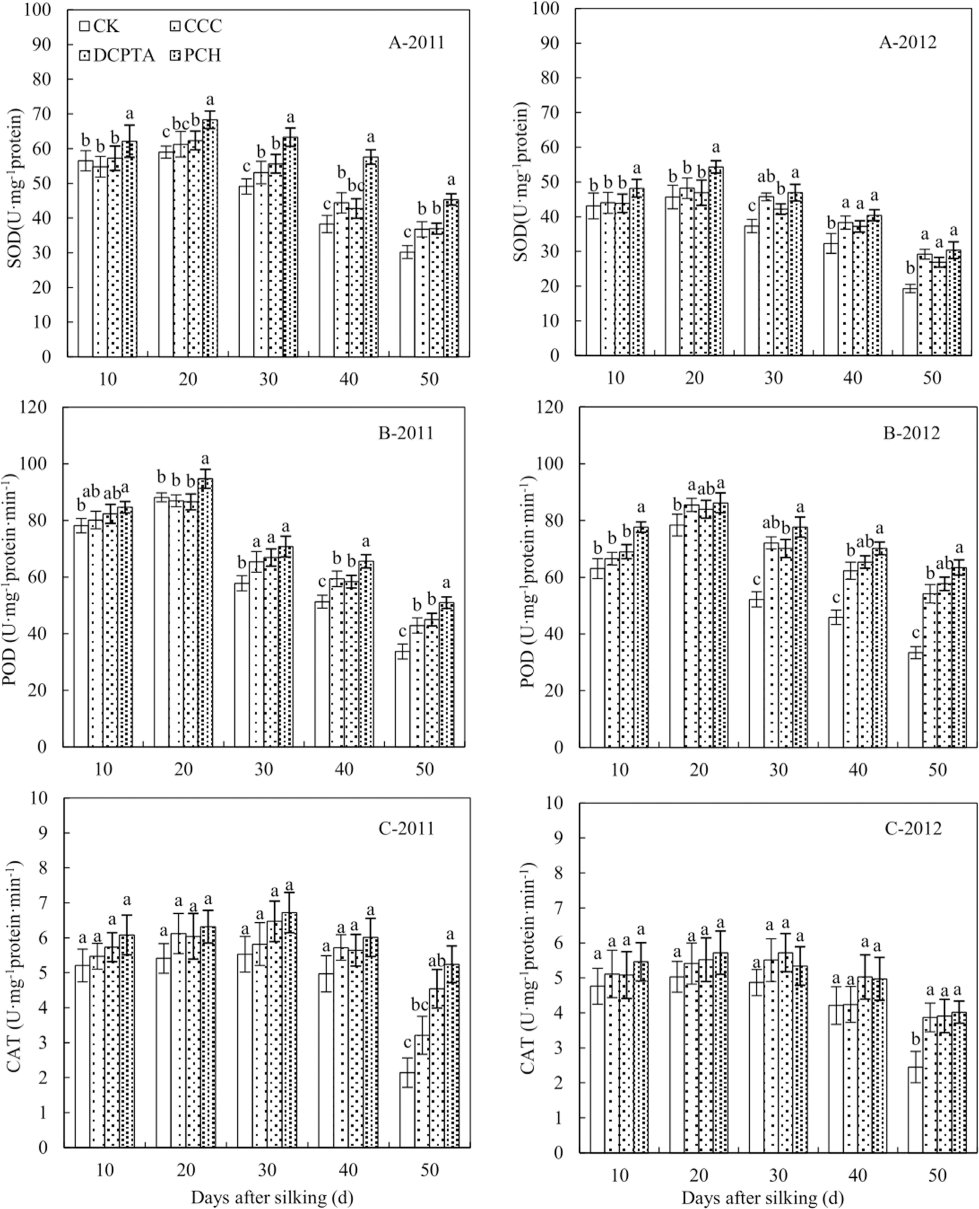
Effect of different plant growth regulators on antioxidant enzyme activity from 2011 to 2012. (A) Superoxide dismutase, SOD. (B) Peroxidase, POD. (C) Catalase, CAT. Values are mean±SD. Same small letter on the bar indicates no significant difference at P<0.05 (LSD test).

Antioxidant enzyme activity represent the anti-aging ability, higher activity can delay leaf senescence. The SOD activity was significantly affected by year, PGRs and sampling time. The interaction of various factors was significant except between three factors (Table 8). From 10 to 20 days after silking, the SOD activity showed an upward trend and a downward trend after that from 2011 to 2012 (Fig 5A). Compared to control, the SOD activity was improved in varying degrees in PGRs-treated plants. Three PGRs could slow the rate of decline in SOD activity. PCH had the best effect for improving SOD activity than CCC and DCPTA in 2011, the difference was significant. The SOD activity displayed a 1.5, 1.2 and 1.2-fold increase in PCH-treated plants, compare to control, CCC and DCPTA at 50 days after silking in 2011(Fig 5A-2011). The SOD activity of 2012 was similar to 2011, PCH showed the best effect for increasing SOD activity than CCC and DCPTA, but the effect was weaker than 2011. There was no significant difference between PCH and CCC, DCPTA at 50 days after silking (Fig 5A-2012)

The POD activity was significantly affected by PGRs and sampling time. The interaction of various factors was significant (Table 8). The POD activity was improved in different PGRs-treated plants. PCH had the best effect for increasing the POD activity from 2011 to 2012 (Fig 5). There was no significant difference between CCC and DCPTA, but it was appeared between PCH and other treatments in 2011 (Fig 5B-2011). The effect of PCH on POD activity in 2012 was weaker than in 2011, significant difference was only appeared between PCH and other treatments at 10 days after silking (Fig 5B-2012).

The CAT activity was significantly affected by year and sampling time. The interaction between year and sampling time was significant (Table 8). The CAT activity was no significant change from 10 to 40 days after silking over the two years, showed a reduction at 50 days after silking in all treatments (Fig 5C). The CAT activity could be increased with different PGRs application, compared to control. From 10 to 50 days, CAT activity of control, CCC, DCPTA and PCH was declined 2, 2.26, 1.19 and 0.84 U·mg^-1^protein·min^-1^ in 2011, PCH-treated plants showed the best result for delaying decreased activity of CAT (Fig 5C-2011). No significant difference was showed between three PGRs treatments, but significant difference was showed between three PGRs treatments and control at 50 days after silking in 2012 (Fig 5C-2012).

**Fig 6:**
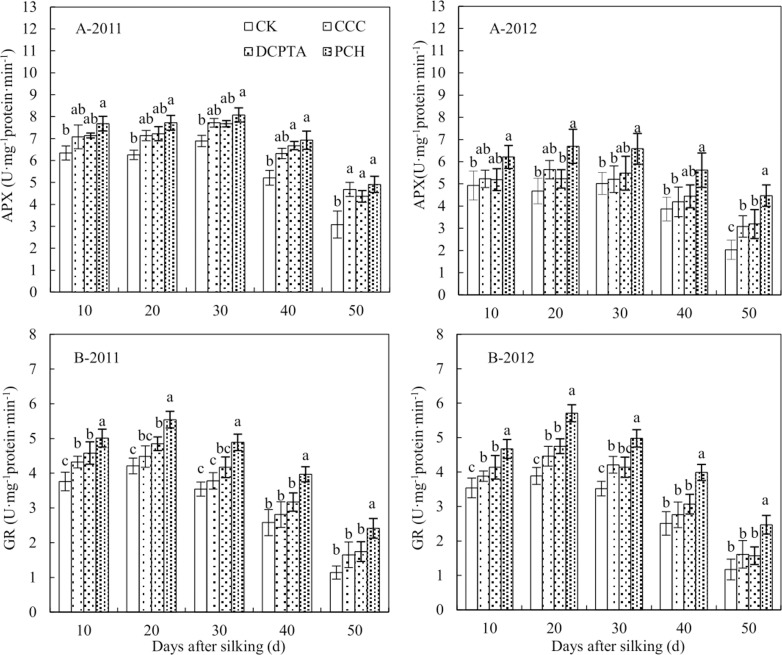
Effect of different plant growth regulators on antioxidant enzyme activity from 2011 to 2012. (A) Ascorbate peroxidase, APX. (B) Glutathione reductase, GR. Values are mean±SD. Same small letter on the bar indicates no significant difference at P<0.05 (LSD test).

The APX activity was significantly affected by year, PGRs and sampling time (Table 8). From 10 to 30 days, the APX activity had a slight rise, declined after that from 2011 to 2012 (Fig 6). With different PGRs application, the APX activity was increased, compared to control. Significant difference was showed between control and three treatments, but was not displayed in three treatments at 50 days after silking in 2011 (Fig 6A-2011). In 2012, PCH-treated plants had the best result for increasing the APX activity (Fig 6A-2012). At 50 days after silking, the APX activity of PCH-treated plants was 2.21, 1.45 and 1.40 times greater than control, CCC and DCPTA.

The GR activity was significantly affected by PGRs and sampling time. The interaction of various factors was not significant (Table 8). GR activity showed the trend after the first increased and then decreased, displayed the highest activity at 20 days after silking from 2011 to 2012 (Fig 6B). The GR activity could be improved in different PGRs-treated plants and PCH showed the best effect. Compared to control, CCC and DCPTA, the GR activity displayed a 2.11, 1.46 and 1.39-fold at 50 days in 2011 (Fig 6B-2011) and 2.12, 1.53 and 1.57-fold in PCH-treated plants in 2012 (Fig 6B-2012). Significant difference was showed between PCH and others at 50 days after silking.

### Membrane Leakage and Lipid Peroxidation

**Fig 7:**
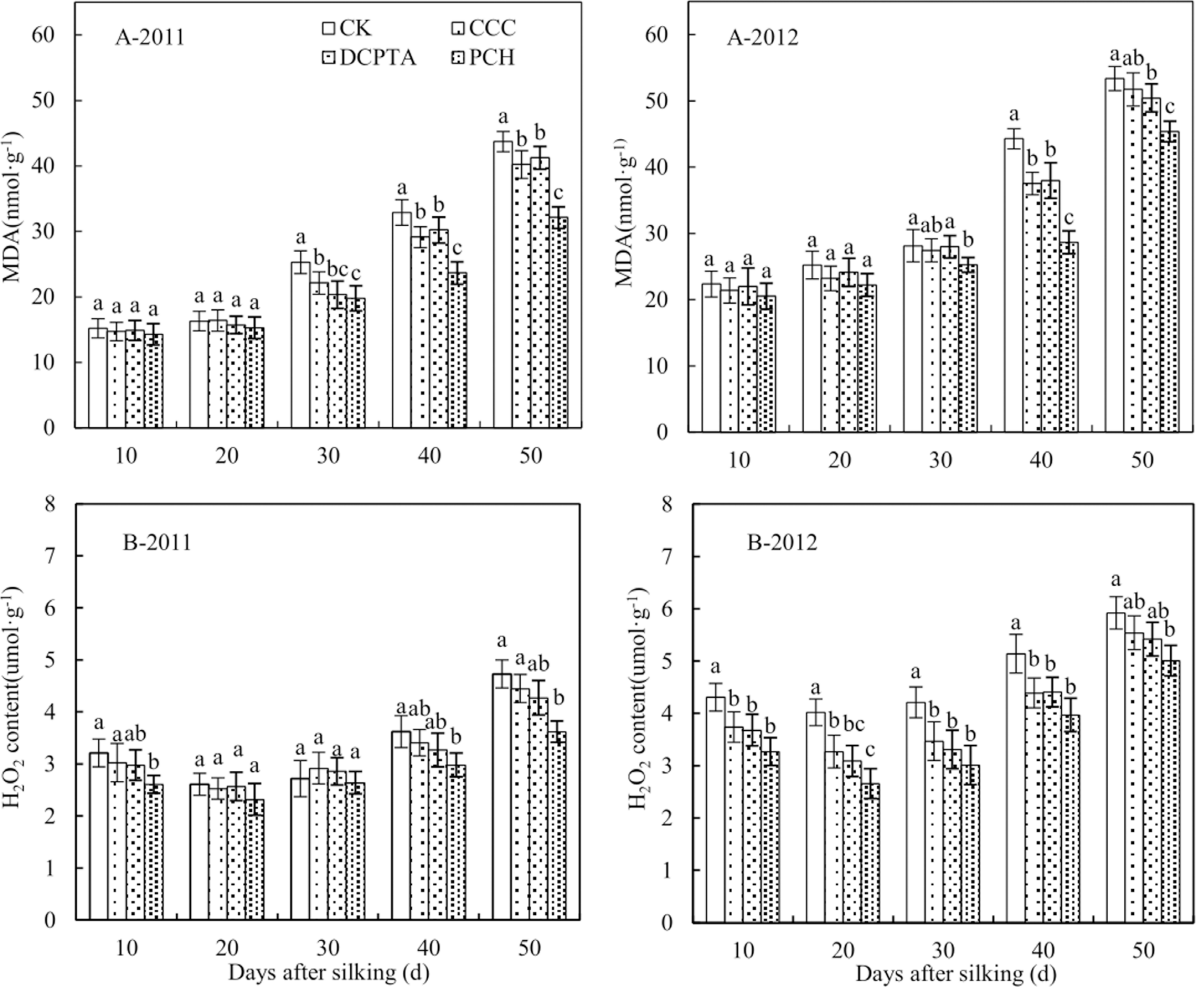
Effect of different plant growth regulators on lipid peroxidation from 2011 to 2012. (A) Malondialdehyde content, MDA. (B) Hydrogen peroxide content, H_2_O_2_. Values are mean±SD. Same small letter on the bar indicates no significant difference at P<0.05 (LSD test).

MDA content was significantly affected by year, PGRs and sampling time. The interaction was significant between year and sampling time. Moreover, the interaction was also significant between PGRs and sampling time (Table 8). MDA content was no significant change from 10 to 20 days after silking over the two years and no significant difference was showed between control and treatments. From 30 to 50 days after silking, MDA content was increased quickly (Fig 7A). MDA content could be declined in varying degrees in PCH-treated plants from 2011 to 2012. It was reduced 26.55% and 14.93% from 2011 to 2012, respectively, compared to control.

Be similar with MDA content, H_2_O_2_ content was also significantly affected by year, PGRs and sampling time. The interaction was significant of various factors except between PGRs and year (Table 8). From 10 to 30 days, H_2_O_2_ content had no significant change, rose quickly after that from 2011 to 2012 (Fig 7B). It could be reduced in PGRs-treated plants and the best effect appeared in PCH-treated plants. H_2_O_2_ content of PCH was lower 1.31, 1.23 and 1.18 times than control, CCC and DCPTA at 50 days after silking in 2011 and the difference was significant between control and PCH, but no significant difference between control and CCC, DCPTA (Fig 7B-2011). Similar results appeared in 2012 (Fig 7B-2012).

**Fig 8:**
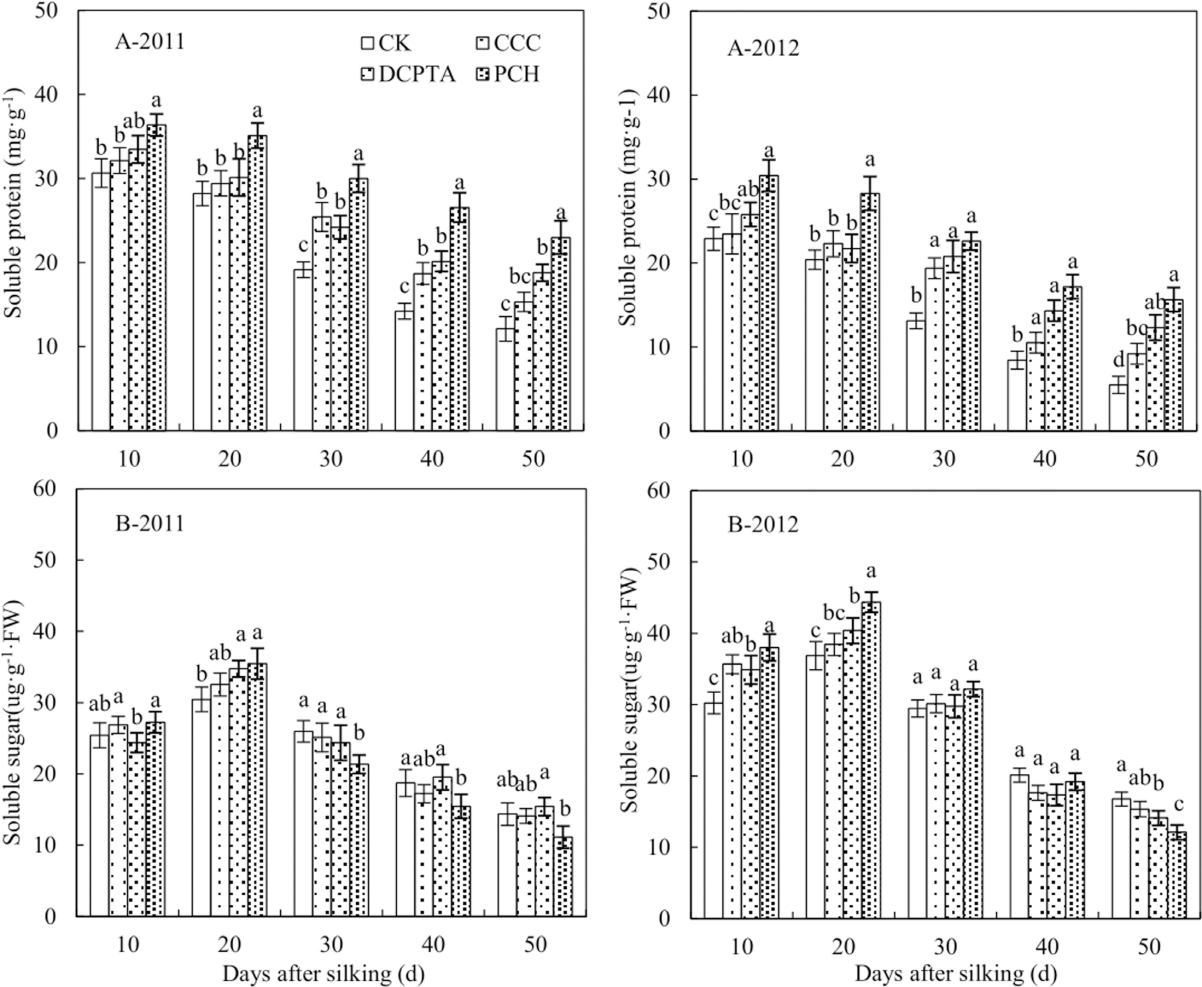
Effect of different plant growth regulators on osmotic substances from 2011 to 2012. (A) Soluble protein content. (B) Soluble sugar content. Values are mean±SD. Same small letter on the bar indicates no significant difference at P<0.05 (LSD test).

With the increase in days, soluble protein content appeared a gradual downward trend from 2011 to 2012 (Fig 8A). Soluble protein content could be increased with different PGRs application and PCH-treated plants displayed higher content than CCC and DCPTA from 2011 to 2012.

Soluble sugar content had a increase from 10 to 20 days after silking, showed a downward trend after that from 2011 to 2012 (Fig 8B). Soluble sugar content of PGRs was higher than control at 20 days after silking, but was lower than control at 50 days from 2011 to 2012. Compared to CCC and DCPTA, PCH-treated plants showed the lowest soluble sugar content over the two years. The declining rate of soluble sugar content was increased in PCH-treated plants and the difference was significant in 2012 (Fig 8B-2012), but no significant difference in 2011 (Fig 8B-2011).

## Discussion

### Chlorophyll content and chlorophyll fluorescence

Chlorophyll is an important pigment in photosynthesis and it plays a role in the absorption and transmission of light energy. At the silking stage, leaves begin to age. The large remobilization for the early stage of leaf senescence is mainly attributed by the degradation of stromal proteins (especially Rubisco) accumulated in chloroplasts [40]. In the aging process, chlorophyll content reduced gradually. As the result, photosynthetic capacity decreased [41]. Some studies had been made in improving plant growth using DCPTA as an exogenous agent in different crop plants. It was well established that DCPTA improved the content of chla, chlb in maize and soybean [42], but the further study showed that the increase of chlorophyll content after the treatment was the result of the volume increase of chlorophyll, not the increasing synthesis of chla and chlb. Be similar with DCPTA, the research showed that the leaf color deepen, leaf thickness increase, chlorophyll content rise, photosynthesis increase in CCC-treated plants [43]. Our results with CCC-DCPTA mixture (PCH) showed likewise an increase of chlorophyll content due to additive effect of CCC and DCPTA. A reasonable explanation of additive effect is DCPTA and CCC increase chlorophyll by diverse regulatory pathways. DCPTA can increase chlorophyll content may be by increasing the volume of chlorophyll and CCC may be by inhibiting synthetic of gibberellin (GA). The physiological basis of the effect is inhibition of the biosynthesis of gibberellin hormone by blocking the formation of ent-Kaurene (GA synthetic precursor) [44]. A reasonable explanation of CCC can block ent-kaurene is affecting enzyme activity from geranylgeranyl pyrophosphate (GGPP) to ent-kaurene [45](*Rademacher 2000*). GGPP is diterpene precursor substances, it can involve in the synthesis of a variety of substances, including carotenoids and chlorophyll. Due to synthesis is hampered from GGPP to ent-kaurene, GGPP may be more conversions to diterpene, and this may be the reason that CCC can increase chlorophyll content.

Photosystem II (*PSII*) is considered as the primary site of injury to the photosynthetic apparatus at stress or senescence process [46]. *PSII* injury can lead to a change in chlorophyll fluorescence. Thus, chlorophyll fluorescence has been used as a powerful and reliable non-invasive method for assessing the changes in the function of *PSII* and for reflecting the primary photosynthetic processes under environmental stress conditions [47, 48]. Some studies showed exogenous agent could improve *PSII* of different plants at stress or senescence process. With regard to the chlorophyll fluorescence parameters, we found that application of 40 mg/L DCPTA on maize seedling caused the increase in *F*_*m*_, *F*_*v*_*/F*_*m*_, *and qP*. The results showed that the light transformation efficiency increased in the reaction center of *PSII* and the primary reaction of photosynthesis was promoted and promoted the excited energy transformation from *LHCII* to *PSII* after spraying optimum concentration DCPTA on plants [49]. The *F*_*v*_*/F*_*m*_ value under optimal growth conditions was around 0.85 in many plants, but it markedly declined during stress. Under drought stress, compared with the control, the *F*_*v*_*/F*_*m*_ value was increased in Me-JA and COR-treated plants. 500mg/L CCC increased *F*_*v*_*/F*_*m*_ and *qP* value in Pistacia chinensis leaves, compared with control, the significant was difference. Our studies were similar to previous studies, DCPTA and CCC could improve chlorophyll fluorescence characteristics in different degrees and the compound mixtures of DCPTA and CCC (PCH) showed better effect. For the regulation mechanism of improving chlorophyll fluorescence of PCH, a reasonable explanation is the increase of chlorophyll content. More chlorophyll molecules can improve the rate of absorption and transmission electron, optimize up-regulated mechanism of *PSII* reaction, and enhance photosynthetic capacity. The improvement of photosynthetic capacity will led to non-photochemical quenching decline, thereby improve chlorophyll fluorescence parameters.

### Enzymatic antioxidants and membrane leakage

ROS are continuously produced by mitochondria, chloroplasts and peroxisomes in higher plants [50–52]. Stress and senescence can break the balance of production and scavenging of ROS, resulting in oxidative damage and lipid peroxidation [53]. MDA is one of the byproducts of lipid peroxidation and its content is routinely used as an indicator of membrane lipid peroxidation [54, 55]. H_2_O_2_ also has a toxic effect on plant cell [56, 57]. Accumulation of enzymatic antioxidants has a substantial role in plant growth and developmental events by regulating ROS production under non stress as well as stress conditions [58–60]. Some results showed that SOD, POD, CAT, APX and GR activity increased greatly in in response to Me-JA in Arabidopsis and peanut seedlings [61, 62]. Exogenously applied spermidine could increase SOD, POD, and CAT activity and reduce MDA content under drought stress in creeping bentgrass. Other studies had shown that DCPTA could reduce the accumulation of MDA, increased SOD and POD activity. Our results were in agreement with previous studies, that is, total activities of antioxidative enzymes increased and the content of MDA and H_2_O_2_ decreased in response to CCC, DCPTA and compound mixtures of DCPTA and CCC (PCH) in maize leaves. Endogenous hormone content can be affected by using CCC and DCPTA in maize leaves. The regulation of endogenous hormones is associated with antioxidant enzyme activity in maize leaf senescence [63]. A reasonable explanation which CCC and DCPTA can affect antioxidant enzyme activity is increasing the relative content of abscisic acid (ABA). ABA shows an important role in increasing the stress resistance of plants, it can promote the generation of new anti-stress protein in plants, induce the recombination of some enzymes. CCC can increase the relative content of ABA by inhibiting the biosynthesis of GA. DCPTA induces carotenoid accumulation and both cis-polyisoprene in guayule and transpolyisoprene in *Eucommia ulmoides*, Oliv.Therefore the mode of action of DCPTA must involve a step early in terpenoid biosynthesis such as the production of mevalonic acid by the enzyme 3-Hydroxy-3-methylglutaryl-CoA reductase [64]. Mevalonic acid is a synthetic precursor of ABA, and DCPTA can increase ABA content by regulating biosynthesis of mevalonic acid in maize leaves. The relative content of ABA may show further increase due to collective effect of CCC and DCPTA in PCH-treated plants, resulting antioxidant enzyme activity increase.

With the accumulation of MDA and H_2_O_2_, the damage of membrane increases, resulting in the changes of membrane permeability. Soluble proteins and sugar can serve as osmotic regulatory agents. They can make cells maintain a comparatively low osmotic potential and resist water stress at high concentrations [65]. Chitosan treatment slightly decreased the rate of soluble protein reduction. Soluble sugar concentration of potato leaves was higher with chitosan treatment than without chitosan at the early stages of drought stress, but there were no obvious differences between treatments at the late stages [66]. Our study were in agreement with previous studies, that is, with the increase of days after silking, soluble protein content decreased, PCH treatment could increase soluble protein content, decrease the rate of decomposition. With the increase of days, soluble sugar showed an upward trend from 10 to 20 days and downward trend after that. PCH treatment was greater than control and other treatments from 10 to 20 days, but lower than control and other treatments after that. The result may be due to PCH promoted transportation of soluble sugar from leaves to kernels at later silking.

### Plant morphology

Plant morphology is one of reasons for affecting maize yield, associate with an effect on lodging resistance, as well as photosynthesis. Shorter and stronger stems have been achieved by using conventional breeding and biotechnology [67], as well as can be obtained by applying PGRs. PGRs can optimize plant morphology and control lodging in maize. Lower lodging percent can decrease mutual overlap in maize leaves, resulting in the improvement of using percent for light. Ethephon (2-chloroethyl phosphonic acid) is a plant growth regulator that inhibits stem elongation and promotes stem thickness, thereby improving plant morphological resistance to lodging. Our results imply that the lodging percent decreased in CCC-treated plants, associated with the reduction of plant height and internode length obviously, as well as the increase of internode diameter and stem strength. But leaves area was reduced in CCC-treated plants, resulting in the decline of photosynthesis area. Leaves area was increased in DCPTA-treated plants, but the lodging percent was increased. Although internode diameter and stem strength were increased in DCPTA-treated plants, the difference was not significant. However, the plant height was significantly increased, resulting the lodging percent was increased in DCPTA-treated plants. Therefore, two type plant growth regulators did not create suitable plant morphology for maize yield. PCH obtained the positive effect of DCPTA and CCC on plant morphology build, decreased plant height and removed the negative effect of CCC on leaf area. CCC can reduce the internode length and plant height by inhibiting biosynthesis of GA, increase the internode diameter and stem strength by regulating biosynthesis of ethylene. Ethylene affects the aligned state of microtubules in stem cells, reduces horizontal arrangement of microtubules, and increases longitudinal arrangement of microtubule. Longitudinal arrangement of microtubule can increase longitudinal deposition of microfibril, limit the magnitude of the cell longitudinal expansion, and promote cell growth toward the transverse direction. DCPTA can promote the production of cytokinin by affecting biological activity of mevalonic. Cytokinin can promote cell division, and this may be the reason that internode length and diameter and stem strength increased by applying DCPTA.

### Grain yield Source-sink relationship and grain yield

Some studies showed that application of ethephon reduces plant height and ear position improving maize resistance to lodging, but lowers yield and grain weight, especially at high application rates [68]. Applying compound mixtures of ethephon and DA-6 increased maize yield, and at all tested plant densities die to improvement of yield components and reduction of lodging by shortening plant height. In our studies, application of EDAH at 7 expanded leaves stage increased yield. The yield increase was mainly caused by a greater kernel number per ear, as well as a greater TKW. Kernel number per ear and TKW increase may be caused by the lodging percentage reduce, photosynthetic capacity increase and anti-aging capacity rise, resulting in the time and rate of matter accumulation increase.

Mason and Maskell put forward crop source-sink theory by researching the distribution of carbohydrates in cotton [69]. Some studies focus on which is the limiting factors of yield between source and sink and three views form gradually: (1) Photosynthetic product is material basis of kernel yield formation and improve source is the main way of increasing yield, (2) After silking stage, yield is proportional to dry matter and total grains. Compare to source, sink capacity shows more important role in yield, (3) It is not comprehensive view that the limiting factors of yield are source or sink. To obtain high-yield, not only the relationship should be coordinated, but also taking into account the coordination of transport [70]. Most previous studies focused on improving the relationship between source and sink, few studies expounded the effect of plant growth regulator on source-sink relationship. In our studies, PCH increased ear size and kernel number per ear and these could be regarded as an increase of sink. PCH improved chlorophyll fluorescence, increased chlorophyll content and leaf area per plant and these could be seen as an increase source. We did not study transport between source and sink, therefore, the effect of PCH on transport could not be expounded. In future study, we should research the effect of PCH on transport and ascertain the regulation that PCH affect source-sink relationship.

## Conclusion

Application of PCH at 7 expandesd leaves stage reduce lodging percent, plant height and internode length, decrease MDA and H_2_O_2_ accumulation, increase internode diameter, stem strength, leaf area and leaf dry weight per plant, improve photosynthetic capacity and antioxidant enzyme activity, resulting in kernel number per ear, thousand kernel weight and grain yield increase.

## Supporting Information

S1 File Supporting Information Data. This file contains date including chlorophyll content, chlorophyll fluorescence, antioxidant enzyme activity, soluble protein and soluble sugar content.
(XLSX)

S2 File Supporting Information Data. This file contains date including yield, ear size, kernel number per ear and thousand kernel weight.
(XLSX)

S3 File Supporting Information Data. This file contains date including plant height, lodging percentage, stem characters, leaf dry weight and leaf area per plant.
(XLSX)
